# Displaced populations due to humanitarian emergencies and its impact on global eradication and elimination of vaccine-preventable diseases

**DOI:** 10.1186/s13031-016-0094-5

**Published:** 2016-11-30

**Authors:** Eugene Lam, Michael Diaz, Allen Gidraf Kahindo Maina, Muireann Brennan

**Affiliations:** 1 Emergency Response and Recovery Branch; Division of Global Health Protection, Center for Global Health; Centers for Disease Control and Prevention, 1600 Clifton Road NE, Atlanta, GA 30309 USA; 2 Department of Global Epidemiology; Rollins School of Public Health, Emory University, Atlanta, GA USA; 3 United Nations High Commissioner for Refugees (UNHCR), Geneva, Switzerland

**Keywords:** Vaccines, Refugees, Poliomyelitis, Measles, Rubella, Disease eradication, Internally displaced persons, Humanitarian emergency, Immunizations, Outbreaks, Civil conflicts, Displacement

## Abstract

Populations affected by humanitarian emergencies may require unique strategies to ensure access to life-saving vaccines and attain sufficiently high population immunity to interrupt virus circulation. Vaccination strategies among displaced populations should not be an afterthought and must be part of the vaccine-preventable disease eradication and elimination initiatives from the start.

According to the UN High Commissioner for Refugees (UNHCR), there were 59.5 million forcibly displaced persons worldwide by the end of 2014 due to persecution, conflict, generalized violence, or human rights violations, and this number is only expected to increase with numerous ongoing global conflicts [1]. These forcibly displaced populations include both refugees who cross international boarders in their escape from conflict, and internally displaced persons (IDPs) who flee conflict but stay within the borders of their own country. The unique challenges of emergency settings often interfere with routine health services and prevent access to recommended vaccinations. This disruption of immunization services increases the number of susceptible individuals and creates a population at particularly high risk for vaccine-preventable diseases (VPDs) targeted for eradication and elimination [2].

There are a number of VPD eradication and elimination initiatives taking place worldwide. Eradication is defined as the permanent reduction to zero of the worldwide incidence of infection caused by a specific agent as a result of deliberate efforts; whereas elimination is the reduction of the incidence of a specified disease to zero in a defined geographic area [3]. Among the eradication and elimination initiatives that are currently in place, perhaps the two most prominent and relevant to emergency settings are the Global Polio Eradication Initiative (GPEI) and the Measles and Rubella Initiative (MRI) [4, 5]. The GPEI launched at the World Health Assembly in 1988 and is a joint partnership between United Nations Children’s Fund (UNICEF), Rotary International, the United States Centers for Disease Control and Prevention (CDC), and the World Health Organization (WHO). The MRI began in 2001 and is a partnership between the American Red Cross, CDC, UNICEF, United Nations Foundation and WHO. With the respective goals of certified global eradication of polio by 2018 [4] and the elimination of measles and rubella in at least five WHO regions by 2020 [5], these programs are among the most promising and ambitious public health endeavors of our time.

Global VPD eradication and elimination initiatives are resource intensive in terms of finance and human resources. Experiences with GPEI and MRI have shown that populations displaced due to humanitarian emergencies can increase the risk of VPD outbreaks and thereby adding to the resources needed to meet VPD eradication and elimination goals [6–10]. Although most outbreaks of polio and measles worldwide do not occur among displaced persons, recent outbreaks among refugees, IDPs, and conflict-affected populations suggest that these vulnerable groups are at risk of transmission of VPDs. The breakdown of immunization services in Syria due to the protracted civil war has caused multiple outbreaks of VPDs, both within the country and the region. The reemergence of polio in Syria for the first time in over 15 years caused concern of re-introduction of polio to neighboring countries into areas with suboptimal vaccination [6]. Outbreaks of measles also increased substantially across Syria and its neighbors including Turkey, Jordan, and Lebanon [10].

The spread of VPDs among displaced populations and susceptible host communities has not been limited to the Middle East. A 2013 outbreak of polio in refugee camps along the Kenya Somalia border spread into the surrounding communities resulting in reactive vaccination efforts in both the camps and non-camp populations [7]. Outbreaks of measles in Dadaab refugee camp in Kenya and Dollo Ado refugee camp in Ethiopia in 2010 and 2011 were linked to the arrival of new refugees from Somalia that was experiencing a measles outbreak during that time [8, 9]. Furthermore, a joint statement by WHO, UNHCR, and UNICEF on refugees, asylum-seekers and migrants entering the WHO European Region has called for the integration of refugee and migrant health into broader national health interventions to ensure equitable and non-discriminatory access to health services, including vaccinations [11]. Due to the dynamic movement and unique characteristics of displaced populations, specific vaccination strategies for such populations at risk of VPD circulation should be examined and documented in light of global efforts for eradication and elimination [12].

Various guidance documents for vaccination efforts in acute humanitarian emergencies are currently available [13, 14] Traditional vaccination strategies among displaced populations have focused on camp settings including vaccination of new arrivals, utilization of transit centers to provide health services including vaccinations, and mass vaccination campaigns [15]. As more displaced persons have moved out of camps and into urban settings [16], different strategies to increase vaccination coverage among non-camp populations are warranted given the unique challenges in locating such populations. To ensure that health services are available to displaced populations who are not in camp setting but integrated into host communities, key partners should provide effective social mobilization and communication to facilitate access to vaccination and other health services along with appropriate language and translation support. Protracted emergencies highlight the need to reinforce routine immunization to prevent repeated large-scale outbreaks of VPDs [17]. Roles and responsibilities of different agencies regarding various aspects of the vaccination programs should be clearly allocated among local and international agencies to capitalize on operational strengths of each partner. Operational guidelines for VPD eradication and elimination efforts should be adapted to the evolving epidemiology of both the disease and affected populations in order to address the unique challenges of reaching displaced populations in humanitarian emergencies [18–20].

Unless plans are made upfront to include displaced populations in national and regional VPD control efforts, global eradication and elimination goals may not be achieved within the targeted time frame. Vulnerable populations affected by humanitarian emergencies may require unique strategies to ensure that these populations have access to life-saving vaccines and attain sufficiently high population immunity to interrupt virus circulation. The Global Vaccine Action Plan (GVAP) 2011–2020 highlights, in its third strategic objective, the importance of ensuring equitable access of vaccines to all people including those in humanitarian crises and conflict zones [21]. GVAP calls for effective communication and establishment of vaccine stockpiles to prevent and respond to VPDs among displaced populations in humanitarian emergencies. If vaccines are to be equitably accessible to all, deliberate efforts to include the most marginalized communities are a must. For this reason, vaccination strategies among displaced populations should not be an afterthought and must be part of the eradication and elimination strategies from the start.

## References

[1] UN High Commissioner for Refugees (UNHCR), UNHCR Mid-Year Trends 2015. 2015. Available at: http://www.refworld.org/docid/568fbb8f4.html . Accessed 12 Mar 2016.

[2] Connolly MA, Gayer M, Ryan MJ, Salama P, Spiegel P, Heymann DL (2004). Communicable diseases in complex emergencies: impact and challenges. Lancet.

[3] Dowdle WR (1998). The principles of disease elimination and eradication. Bull World Health Organ.

[4] Global Polio Eradication Initiative, Polio Eradication & Endgame Strategic Plan 2013–2018. 2013. Available at: http://www.polioeradication.org/Portals/0/Document/Resources/StrategyWork/PEESP_EN_US.pdf. Accessed 12 Mar 2016.

[5] World Health Organization, Global Measles and Rubella Strategic Plan 2012–2020. 2012. Available at: http://apps.who.int/iris/bitstream/10665/44855/1/9789241503396_eng.pdf. Accessed 13 Mar 2016.

[6] Eichner M, Brockmann SO (2013). Polio emergence in Syria and Israel endangers Europe. Lancet.

[7] Combined use of inactivated and oral poliovirus vaccines in a large-scale campaign in refugee camps and host communities - Kenya, December 2013. Wkly Epidemiol Rec, 2014;89(12): 127–32 24707518

[8] Navarro-Colorado C, Mahamud A, Burton A, Haskew C, Maina GK, Wagacha JB (2014). Measles outbreak response among adolescent and adult Somali refugees displaced by famine in Kenya and Ethiopia, 2011. J Infect Dis.

[9] Polonsky JA, Ronsse A, Ciglenecki I, Rull M, Porten K (2013). High levels of mortality, malnutrition, and measles, among recently-displaced Somali refugees in Dagahaley camp, Dadaab refugee camp complex, Kenya, 2011. Confl Health.

[10] Sharara SL, Kanj SS (2014). War and infectious diseases: challenges of the Syrian civil War. PLoS Pathog.

[11] World Health Organization, UN Children's Fund, and UN High Commissioner for Refugees, WHO-UNHCR-UNICEF Joint statement on general principles of vaccination of refugees, asylum-seekers and migrants in the WHO European Region. 2015. Available at:http://reliefweb.int/sites/reliefweb.int/files/resources/EuropeVaccinationPosition_WHO-UNHCR-UNICEFNov.pdf. Accessed 4/9/2016.

[12] Minetti A, Bopp C, Fermon F, François G, Grais RF, Grout L (2013). Measles outbreak response immunization is context-specific: insight from the recent experience of Médecins Sans Frontières. PLoS Med.

[13] Sphere Project, Sphere Handbook: Humanitarian Charter and Minimum Standards in Disaster Response, 2011. 2011. Available at: http://www.refworld.org/docid/4ed8ae592.html. Accessed on 13 Mar 2016.

[14] World Health Organization, SAGE working group on vaccination in humanitarian emergencies : vaccination in acute humanitarian emergencies: a framework for decision making. 2013. Available at: http://www.who.int/iris/handle/10665/92462. Accessed 13 Mar 2016.

[15] Kamugisha C, Cairns KL, Akim C (2003). An outbreak of measles in Tanzanian refugee camps. J Infect Dis.

[16] Spiegel PB (2010). Health-care needs of people affected by conflict: future trends and changing frameworks. Lancet.

[17] Mancini S, Coldiron ME, Ronsse A, Ilunga BK, Porten K, Grais RF (2014). Description of a large measles epidemic in Democratic Republic of Congo, 2010–2013. Confl Heal.

[18] World Health Organization, Planning and Implementing High Quality Supplementary Immunization Activities for Injectable Vaccines using an example of Measles and Rubella Vaccines. A Field Guide. 2016. Available at: http://www.who.int/immunization/diseases/measles/SIA-Field-Guide-revised.pdf. Accessed on 8/3/2016.

[19] World Health Organization, Measles SIAs Planning and Implementation Field Guide. Regional Office for Africa (AFRO). 2010. Available at: http://www.measlesrubellainitiative.org/wp-content/uploads/2013/06/WHO-AFRO-Measles-Fieldguide-April-2011.pdf. Accessed on 8/3/2016.

[20] World Health Organization, Measles Elimination Field Guide. Regional Office for the Western Pacific (WPRO). 2013. Available at: http://www.wpro.who.int/immunization/documents/measles_elimination_field_guide_2013.pdf. Accessed on 8/3/2016.

[21] World Health Organization, Global vaccine action plan 2011–2020. 2013. Available at: http://www.who.int/immunization/global_vaccine_action_plan/en/. Accessed 16 Mar 2016.

